# Physical activity and competitive sport safety for children affected by inherited cardiac conditions and selected acquired cardiomyopathies: emerging evidence and areas for further inquiry

**DOI:** 10.1007/s00431-026-06768-y

**Published:** 2026-03-04

**Authors:** Scott Kendall, Andrea Greco, Nicoletta Cantarutti, Andreia Constante, Daniel Diaz-Gil, Simon D’hulst, Roger Esmel-Vilomara, José Luis López-Guillén, Katarzyna Luczak-Wozniak, Gian L. Ragazzoni, Wannes Renders, Iulia Rodoman, Christophe Vô, Sara Moscatelli, Terrence Prendiville, Pascal McKeown, Georgia Sarquella-Brugada, Frank Casey

**Affiliations:** 1 Wellcome Wolfson Institute for Experimental Medicine, Queen’s University Belfast, 97 Lisburn Road, Belfast, BT9 7BL UK; 2 Arrhythmias, Inherited Cardiac Diseases and Sudden Death Unit, Hospital Sant Joan de Déu, Esplugues de Llobregat, 08950 Spain; 3 Arrítmies Pediàtriques, Cardiologia Genètica i Mort Sobtada, Malalties Cardiovasculars en el Desenvolupament, Institut de Recerca Sant Joan de Déu (IRSJD), Esplugues de Llobregat, 08950 Spain; 4 Pediatrics Department, School of Medicine, Universitat de Barcelona, Barcelona, 08036 Spain; 5 Pediatric Cardiology and Arrhythmias Unit, Bambino Gesù Children’s Hospital, IRCCS, Rome, Italy; 6 Centro Clínico Académico de Lisboa, Clínica Universitária de Cardiologia Pediátrica, Lisbon, Portugal; 7 Pediatric Cardiology Department, Reference Center for Congenital Heart Diseases, Hospital de Santa Marta, Unidade Local de Saúde São José EPE, Lisbon, Portugal; 8 Department of Pediatrics, Harvard Medical School, Boston, MA USA; 9 Department of Cardiology, Boston Children’s Hospital, Boston, MA USA; 10 Division of Pediatric Cardiology, Department of Pediatrics, Ghent University Hospital, Corneel Heymanslaan 10, Ghent, 9000 Belgium; 11 Faculty of Medicine, Universitat Autònoma de Barcelona, Barcelona, Spain; 12 Pediatric Cardiology, Vall d’Hebron Hospital Campus, Barcelona, Spain; 13 Pediatric Cardiology, Hospital de la Santa Creu i Sant Pau, Sant Pau Research Institute (IR Sant Pau), Barcelona, Spain; 14 Division of Cardiology, Labatt Family Heart Centre, The Hospital for Sick Children, University of Toronto, Toronto, Canada; 15 Department of Pediatric Cardiology and General Pediatrics, Medical University of Warsaw, 61 Zwirki i Wigury Street, Warsaw, 02–091 Poland; 16 Sports Cardiology and Rehab Unit, Department of Medical Biotechnologies, University of Siena, Siena, Italy; 17 Pediatric Cardiology Unit, Human Molecular Genetics Laboratory, Institute of Mother and Child, Chisinau, Republic of Moldova; 18 Division of Pediatric Cardiology, Department of Pediatrics, Cliniques Universitaires Saint-Luc, 10, Avenue, Hippocrate, Brussels 1200 Belgium; 19 Centre for Inherited Cardiovascular Diseases, Great Ormond Street Hospital, London, WC1N 3JH UK; 20 Institute of Cardiovascular Sciences, University College London, London, WC1E 6BT UK; 21 Paediatric Cardiology Department, Royal Brompton and Harefield Hospitals, Guy’s and St. Thomas’ NHS Foundation Trust, London, SW3 5NP UK; 22 Children’s Health Ireland at Crumlin, Cooley Rd, Drimnagh, Dublin D12 N512 Ireland; 23 School of Medicine, Dentistry and Biomedical Sciences, Queen’s University Belfast, 97 Lisburn Road, Belfast, BT9 7BL UK; 24 European Reference Network for Rare, Low Prevalence and Complex Diseases of the Heart-ERN GUARD-Heart, Amsterdam, The Netherlands

**Keywords:** Physical activity, Inherited cardiac conditions, ICC Cardiomyopathy Long Qt Syndrome Marfan's syndrome

## Abstract

Physicians tasked with caring for children with inherited cardiac conditions (ICCs) face complex decisions regarding safe participation in physical activity and competitive sport. Historically, concerns over sudden cardiac death (SCD) and disease progression led to widespread exercise restrictions. However, emerging evidence and evolving guidelines now support a more nuanced, risk-based approach. This narrative review explores current recommendations for exercise in children with major ICCs—including channelopathies, cardiomyopathies, and aortopathies. It highlights advances in risk stratification, the use of exercise testing, cardiac imaging, and genetic information to guide individualised recommendations. Shared decision-making (SDM) is emphasised as central to balancing arrhythmic and disease progression risk with the substantial physical, psychological, and social benefits of exercise. The review also discusses non-inherited conditions like anthracycline-induced cardiomyopathy and myocarditis. Despite progress, substantial variation exists in international guidelines, and there remains a paucity of paediatric-specific data. This poses a significant challenge and often necessitates utilising adult data when making decisions for children.

* Conclusion*: Further research, including registries and multi-centre studies, is essential to improve evidence-based recommendations and empower clinicians, patients, and families in decision-making. A personalised approach—grounded in expertise, communication, and longitudinal follow-up—is critical to safely supporting children with ICCs in leading active, fulfilling lives.

**Graphical Abstract:**

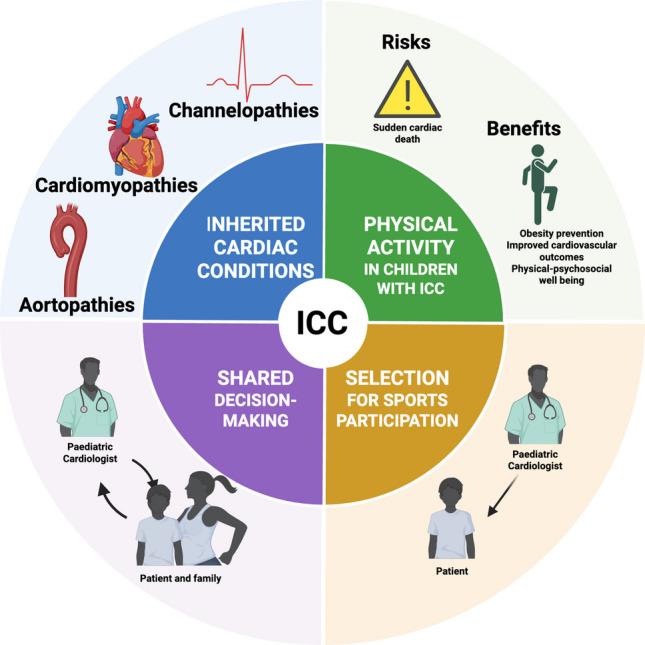

**Supplementary Information:**

The online version contains supplementary material available at 10.1007/s00431-026-06768-y.

**Table:** 

**What is Known:** *• Inherited cardiac conditions (ICCs) are associated with ventricular arrhythmia, heart failure, and sudden cardiac death (SCD) prompting caution around sports and physical activity.* *• Myocarditis and anthracycline-induced cardiomyopathy are common aetiologies of cardiac dysfunction.*
**What is New:** *• Observational studies in paediatric cohorts are reassuring for continued participation in sports following guideline-directed shared decision-making.* *• Cardiac rehabilitation improves left ventricular function in paediatric cancer survivors.*

## Introduction

Inherited cardiac conditions (ICCs) are a diverse group of genetic disorders affecting the heart. They are commonly categorised into three main categories: channelopathies, cardiomyopathies, and aortopathies.

Channelopathies involve abnormalities in ion channels that regulate the cardiac action potential, predisposing individuals to ventricular arrhythmias (VAs) and sudden cardiac death (SCD). Major examples include congenital long QT syndrome (LQTS), Brugada Syndrome (BrS), and catecholaminergic polymorphic ventricular tachycardia (CPVT) [1].

Cardiomyopathies (CM) affect the structure and function of cardiac myocytes and can lead to heart failure, VAs, and SCD, depending on the specific pathology. In the paediatric population, the most common forms are dilated cardiomyopathy (DCM) and hypertrophic cardiomyopathy (HCM) [2, 3]. DCM is the leading indication for paediatric heart transplantation [4]. This review will also consider two non-inherited causes of CM: anthracycline exposure [5] and myocarditis [6] due to phenotypic overlap and relatively high incidence in clinical practice [5, 6]. Less common forms of cardiomyopathy such as arrhythmogenic right ventricular cardiomyopathy (ARVC), non-dilated left ventricular cardiomyopathy (NDLVC), and restricted cardiomyopathy (RCM) are not discussed in this article due to paucity of paediatric data on exercise in these conditions.

Aortopathies/heritable thoracic aortic disease (HTAD) are connective tissue disorders that result in aortic dilatation, which, if untreated, can lead to aneurysm and dissection. Examples include Marfan syndrome and Loeys-Dietz syndrome (LDS) [7].

Given the significant risks associated with ICCs, children have traditionally been subject to restrictions on physical activity and sports participation [8]. However, this restrictive approach is not without its own risks. A sedentary lifestyle can contribute to obesity and worsening cardiovascular outcomes [9, 10], while also depriving children of the physical, psychological, and social benefits of sport [11]. The definition of an athlete is someone who engages in regular exercise and training with an emphasis on performance and sports competition. There is a spectrum ranging from casual leisure-time athletes to professional “elite” athletes [8, 12, 13].

Paediatric cardiologists must help patients and their families navigate these complex decisions, balancing medical risk with individual aspirations through a shared decision-making (SDM) approach [14]. Over the past two decades, progress in risk stratification, therapy, and guidelines has supported a more permissive stance in selected patient groups, allowing greater participation in sports for selected children with ICCs [15]. This article aims to summarise the current evidence surrounding physical activity in children with ICCs, providing a useful resource for clinicians advising families as well as highlighting some of the differences noted between advisory bodies. It will frequently cite sporting guidelines produced for adults, which, in the absence of robust paediatric data, are a necessity for paediatric cardiologists. This highlights the need for dedicated longitudinal paediatric studies in all conditions dealt with in this article.

## Channelopathies

This section describes the current guidelines for the channelopathies, including recent research which has led to significant liberalisation of exercise policy, particularly in LQTS.

### Congenital long QT syndrome (LQTS)

LQTS is a group of monogenic conditions associated with delayed myocardial repolarisation, increasing the risk of VA. There are three major types (LQT1, LQT2, and LQT3) corresponding to variants in the genes KCNQ1, KCNH2, and SCN5A, respectively. Variants in these three genes account for 75–90% of LQTS cases [16]. Retrospective studies investigating cardiac arrest during sports participation have identified LQTS as a significant risk factor, particularly LQT1 [17]. For decades, this led to the blanket exclusion of individuals with LQTS from competitive sports. However, this approach has evolved considering observational data showing low event rates and questioning the assumption that physical activity substantially increases risk [15, 18–20]. A recently published observational study enrolling 1400 participants, including 293 athletes aged 8–22 years, compared outcomes in patients with LQTS who engaged in physical activity versus those who did not and found no statistically significant difference in arrhythmic events between athletes and non-athletes [21]. All children with LQTS should undergo pre-participation screening in the form of resting ECG and exercise stress testing to stratify their risk. Medication doses should be optimised utilising exercise stress data [22]. In cases of recurrent symptoms or intolerance to medication, left cardiac sympathetic denervation (LCSD) may be considered [22]. Personal possession of an automated external defibrillator is a contentious point advocated for by the American Heart Association/American College of Cardiology (AHA/ACC) but not recommended by the European Society of Cardiology (ESC) [23]. For all forms of genetically proven LQTS, the ESC advises against competitive sports if the QT interval exceeds 470 ms or if an implantable cardioverter-defibrillator (ICD) has been implanted; in genotype positive, phenotype negative individuals (QTc < 470 ms in men and < 480 ms in women), competitive sports can be considered following a risk assessment balancing psychological harm of not competing with arrhythmic risk [13]. Meanwhile, the AHA/ACC advise it is permissible for LQTS patients with a QT interval > 470 ms who are under expert led care to continue in competitive sports, following guideline directed therapy, a risk assessment and a documented emergency action plan [23]. In LQT1 patients, caution is required as it is considered the highest risk for VA during sports and especially swimming. The ESC precludes LQT1 patients from swimming or diving, and the AHA/ACC permit swimming and diving in these patients if precautions (not training alone/automated external defibrillator AED available) are taken. Given the mortality associated with drowning [24] far exceeds that of LQTS, it would seem prudent that all affected individuals learn to swim, even if they decided to pursue other sports competitively. There are no sporting recommendations specific to LQT2 or LQT3 patients. Any exercise prescription made must consider disease severity, medication adherence, setting and competitive level of sport being pursued; affected individuals exercising alone is not prudent [25].

### Brugada syndrome (BrS)

BrS is a condition characterised by coved ST elevation pattern either on the resting ECG or on provocation testing; numerous questions still exist regarding its pathophysiology (including the role of genetics), diagnosis, risk stratification, and management [26]. Exercise restriction is no exception, as the relationship between BrS and SCD, and physical activity remains uncertain. SCD in BrS typically occurs at rest and is not catecholamine-sensitive [27]. Exercise activates the sympathetic nervous system, which generally suppresses arrhythmogenesis in BrS. However, increased vagal tone during the early recovery phase of exercise may unmask the BrS ECG pattern [28], trigger premature ventricular contractions (PVCs) [29], or even induce VAs. Despite these observations, no direct causal link has been demonstrated between exercise and SCD in BrS [22]. Over the past two decades, adult guidelines have evolved from systematic exclusion from competitive sports [8] to a more nuanced position acknowledging that there is no compelling evidence to support universal exercise restriction [22, 23]. Participation should be based on an SDM process following comprehensive individual assessment. In asymptomatic patients without high-risk features, competitive sports can generally be permitted with appropriate precautions. Any exercise prescription should include avoidance of certain triggers such as specific drugs, heavy meals, and overheating (especially in prolonged endurance events) [22]. However, individuals with established risk factors—such as a history of arrhythmic symptoms, a spontaneous type 1 ECG pattern, and abnormal cardiac imaging (given the potential overlap between BrS and arrhythmogenic cardiomyopathy)—should be counselled more cautiously, and competitive sports may need to be avoided and delayed for 3 months in the case of ICD insertion [13].

### Catecholaminergic polymorphic ventricular tachycardia (CPVT)

CPVT is a rare inherited arrhythmia syndrome characterised by adrenergic mediated VAs, most often triggered by physical exertion or emotional stress [30]. Due to this strong association between sympathetic stimulation and arrhythmogenesis, international guidelines have historically recommended strict avoidance of vigorous exercise and competitive sports in all patients with CPVT [31]. Non-selective beta-blockers (particularly nadolol) remain the mainstay of treatment; flecainide is often added when arrhythmias persist and to better control persistent VAs, early LCSD may be considered. Exercise stress testing is essential both at diagnosis and during follow-up (every 6 12 months) to assess arrhythmic burden and treatment efficacy [30]. A recent single-centre study found that sports in CPVT was associated with earlier diagnosis in childhood, likely due to more events. The data also suggested that in highly selected cases, specifically those without exercise-induced arrhythmias on repeated testing and excellent adherence to therapy, continuing play may well carry a comparable risk to affected individuals not taking part in sports [32]. The Heart Rhythm Society (HRS) and AHA/ACC both advise that return to play (RTP) may be considered after thorough evaluation and SDM [22, 23]. The general recommendation remains that competitive sports should be avoided if during exertion arrhythmias are not fully suppressed (ventricular couplets or more extensive non-sustained ventricular tachycardia) and aggressive treatment including triple therapy (flecainide, beta-blocker, and LCSD) may be necessary. Exercise prescriptions in CPVT must be cautious; it is one of the few inherited arrhythmia syndromes in which exercise is a clear trigger for life-threatening events. A carefully individualised approach can allow some patients to remain active under expert guidance. The ESC continues to advise against competitive sports and strenuous activity [33]. See Table 1 below for a suggested work up, contraindications, and follow-up.

**Table 1:** Suggested recommendations to allow sports participation, contraindications, and follow-up in channelopathies (adapted from ESC guidelines on sports cardiology and exercise in patients with cardiovascular disease) [13]

Condition	Assessment before sport	Sport contraindication	Follow-up
LQTS	ECG Holter monitoring Exercise test	• QTc > 500 ms • Genetically confirmed with QTc ≥ 470 ms in men or ≥ 480 ms in women • LQTS1 should not engage in sports that include diving in cold water **(contested)** • Prior cardiac arrest or arrhythmic syncope	Repeat exercise testing
Brugada syndrome	ECG (high lead) Holter monitoring Exercise test	Sports/conditions that increase core temperature > 39 °C	
CPVT	Exercise test (including burst protocol in genotype (+) phenotype (−))	Symptomatic CPVT	Repeat exercise test every 6–12 months

*CPVT*, catecholaminergic polymorphic ventricular tachycardia; *ECG*, electrocardiograph; *LQTS*, long QT syndrome; *LQTS1*, long QT syndrome type 1; *QTc*, corrected QT interval

## Cardiomyopathies (CM)

Cardiomyopathies (CM) represent a significant subset of inherited cardiac conditions implicated in SCD among young individuals [34]. The well-established association between exercise and SCD [13, 23, 35] in this population, along with evidence suggesting that physical activity may accelerate disease progression in certain phenotypes, has historically led to broad exercise restrictions for affected individuals [12, 35, 36]. These recommendations were traditionally applied uniformly, irrespective of the specific cardiomyopathy subtype or disease severity. However, the growing recognition of the heterogeneity in disease expression has shifted clinical perspectives toward a more individualised approach [13, 23, 34, 35], particularly in paediatric populations, where the psychosocial and developmental benefits of physical activity must be carefully balanced with cardiovascular risk [37]. The advancements in risk stratification and increasing observational data now provide a more comprehensive approach to determining the safety of physical activity in children with some forms of CM; however, there is still a lack of paediatric data in rarer forms. This section explores current evidence surrounding physical activity and sports participation in children with CM.

### Hypertrophic cardiomyopathy

Hypertrophic cardiomyopathy is characterised by hypertrophy of the left ventricle variably causing left ventricular outflow tract (LVOT) obstruction, systolic and diastolic dysfunction, and ventricular arrhythmia [35]. The ESC and the AHA/ACC guidelines provide distinct perspectives on physical activity in the management of patients with HCM [13, 23] (see Table 2).

**Table 2:** Suggested recommendations to allow sports participation, contraindications, and follow-up in cardiomyopathies (adapted from ESC guidelines on sports cardiology and exercise in patients with cardiovascular disease) ([13]

Condition	Assessment before sport	Sport contraindication	Follow-up
HCM	Holter monitoring preferably 48 h Echocardiography Exercise echocardiography (if symptomatic during exercise, no LVOTO, but SAM present) CMR Exercise test	• High-intensity exercise contraindicated when risk factors present (cardiac symptoms or history of cardiac arrest or unexplained syncope; Risk calculator score (≥ 4%); LVOT gradient at rest > 30 mmHg; abnormal BP response to exercise; exercise-induced arrhythmias; extensive LGE ≥ 15%, possibly highly dynamic sports) • Sports where occurrence of syncope may be associated with harm or death	6 monthly in adolescent who are more vulnerable to exercise related SCD Annual assessment for genotype positive phenotype negative
DCM	Echocardiography Exercise testing Holter ECG CMR Genotype	• LMNA, FLNC, TMEM43, DSP mutations • symptoms or history of cardiac arrest or unexplained syncope • LVEF < 45% • ventricular arrythmia • LGE > 20% on CMR	6 months in adolescents and LMNA/FLNC mutations
Acute myocarditis	Echocardiography Cardiac biomarkers Holter ECG Exercise test Consider CMR	• Acute phase (< 3–6 months) • LV dysfunction • Complex arrhythmias • Extensive myocardial scar (> 20% LGE)*	Periodic reassessment is advised, especially within the first 2 years after diagnosis

*BP*, blood pressure; *CMR*, cardiac magnetic resonance; *DCM*, dilated cardiomyopathy; *DSP*, desmoplakin; *ECG*, electrocardiograph; *EF*, ejection fraction; *ESC*, European Society of Cardiology; *FLNC*, Filamin C; *HCM*, hypertrophic cardiomyopathy; *LGE*, late gadolinium enhancement; *LMNA*, Lamin A/C; *LQTS*, long QT syndrome; *LV*, left ventricle; *LVOT*, left ventricular outflow tract; *LVOTO*, left ventricular outflow tract obstruction; ms, milliseconds; nsVT, non-sustained ventricular tachycardia; *QTc*, corrected QT interval; *SAM*, systolic anterior motion TMEM43, Transmembrane Protein 43

The AHA/ACC adopts an individualised approach that emphasises SDM, integrating mental health and quality-of-life considerations into the risk–benefit analysis [23, 38]. This scientific statement explicitly supports participation in moderate-to-vigorous exercise for carefully selected, low-risk individuals and does not consider the presence of an ICD to be an absolute contraindication to sports practice [38]. This approach has recently been supported by the results of a large observational study (including 221 participants who were children) comparing sedentary HCM patients with those exercising vigorously, and there was no significant difference in event rate between groups [39].

The ESC guidelines acknowledge the uncertainty regarding the effects of sports on HCM and advise that competitive sports are permissible in genotype-positive, phenotype-negative patients and in those with a mild phenotype. Individuals with high-risk features are advised not to compete. Furthermore, several key factors should be considered before recommending the type and intensity of the activity, including (a) the presence of symptoms, (b) a calculated risk score, (c) the presence of resting or inducible LVOT obstruction during exercise, (d) an abnormal blood pressure response to exercise, and (e) the occurrence of arrhythmias at rest or during exertion. Notably, recommendations are predominantly based on adult data, and there is limited evidence specifically addressing paediatric populations [13, 35]. Both the ESC and the AHA state that genotype-positive, phenotype-negative individuals may participate in competitive sports of any intensity, citing a very low risk of cardiac events, provided there is regular clinical review and individualised decision-making based on family history and risk tolerance [23, 35]. Many recommendations for treatment and exercise prescription in paediatric HCM is guided by evidence-based tools. The HCM Risk-Kids [40, 41] model provides a validated method to estimate the 5-year SCD risk in children, using clinical parameters such as maximal wall thickness, unexplained syncope, and non-sustained ventricular tachycardia. In parallel, PRIMACY, another risk model, places greater emphasis on arrhythmic burden and device therapy endpoints to guide clinical decisions such as ICD implantation [42]. Additionally, emerging pharmacologic therapies such as myosin inhibitors (e.g. Mavacamten), offer promising improvements in cardiac physiology and exercise tolerance, potentially widening the scope of safe physical activity in select patients [43]. Mavacamten has been approved in adults with HCM with a trial in adolescents on-going at the time of writing [44]. Another potential useful tool is exercise stress testing, in children with HCM exercise induced ischaemia and abnormal blood pressure results have both been associated with increased SCD risk [45].

### Dilated cardiomyopathy

DCM is defined by ventricular dilatation, mainly of the left ventricle (LV), and systolic dysfunction. Phenotypic expression of DCM spans from progressive symptoms of heart failure, with severe impairment of left ventricular ejection fraction (LVEF), to limited echocardiographic or ECG abnormalities. Ventricular arrhythmias, potentially leading to SCD, are often present in DCM, although these are less frequent in children [46–48]. Regarding the paediatric population, specific recommendations remain scarce and most clinical practice is based on extrapolation of adult cohorts. Specific considerations include the following: aetiological work-up, including genetic testing, optimised treatment should be established if high-intensity exercise or competitive sport participation is requested, cardiac magnetic resonance imaging (CMR) evaluation should be considered prior to the SDM process, given the increased risk for VA and SCD in the presence of fibrosis [49, 50]. Current guidelines mainly rely on symptomatology (incl. arrhythmias) and LVEF, highlighting the importance of thorough clinical evaluation of individuals requesting exercise advice. This evaluation needs to ascertain the aetiology, assess clinical status, LV function, and dilation, and assess exercise-induced symptoms or arrhythmias. In general, competitive sports and high- to very high–intensity sports need to be avoided in symptomatic patients or when LVEF ≤ 40% [13, 23, 35]. Underlying genotype plays a significant role in risk stratification, given that certain genes are by themselves associated with arrhythmias, regardless of DCM manifestation [51]. However, only 30–40% of paediatric patients have a causative variant identified [51, 52]. Guidelines on genotype-positive, phenotype-negative patients are rather pragmatic in allowing high-intensity exercise and competitive sport participation, since the available evidence on disease progression is limited [13, 23, 35]. Importantly, excluding patients with a pathogenic variant in specific genes (Lamin A/C (LMNA) [53], Filamin C (FLNC)-truncating variants [54], and Transmembrane 43 (TMEM43)) is likely warranted; whether this also applies to the other high-risk genotypes (Phospholamban (PLN), Desmoplakin (DSP), RNA-binding motif protein 20 (RBM20)) remains inconclusive [35]. In addition, evidence of the association of exercise with worse LV systolic function and higher prevalence of AF in LMNA positive patients is present [51]. It is currently unknown if this association is also present in other high-risk genotypes, so prudence is warranted. Specific tools for supporting decision making regarding ICD implantation for adults are available [55, 56]; similar models for children are clearly desirable.

### Arrhythmogenic right ventricular cardiomyopathy (ARVC)/arrhythmogenic cardiomyopathy (ACM)

Arrhythmogenic right ventricular cardiomyopathy (ARVC) was originally characterised by right ventricular dysfunction, fibrofatty myocardial replacement, characteristic ECG changes, ventricular arrhythmias of right ventricular origin, and pathogenic variants in desmosomal genes [35]. Recognition that the left ventricle may also be significantly involved has led to the adoption of the broader term arrhythmogenic cardiomyopathy (ACM) by the American Heart Association [23], although the European Society of Cardiology continues to use the ARVC nomenclature [35]. Children and adolescents with ACM have a markedly elevated risk of ventricular arrhythmias and sudden cardiac death during vigorous physical activity. Current guidelines and emerging evidence support avoidance of competitive and high-intensity sports in children with ACM, while cautiously allowing supervised low-to-moderate-intensity activity in selected cases following individualised risk assessment [23, 35]. Experimental and clinical studies demonstrate that intense exercise accelerates fibrofatty myocardial replacement and increases arrhythmogenic risk [57, 58]. Earlier AHA/ACC and HRS guidance recommended complete avoidance of competitive sports [59]. Updated 2025 recommendations specify strong restriction for plakophilin-2 (PKP2) mutation carriers, while evidence remains insufficient to mandate universal disqualification for other genotypes or genotype-negative, phenotype-positive individuals, who instead require close follow-up [23]. Registry studies show worse ventricular function and exercise capacity in patients continuing high-intensity exercise [60]. Overall, the guidelines emphasise exercise restriction, careful monitoring, and thorough preparticipation screening to reduce arrhythmic risk and disease progression.

## Anthracycline cardiomyopathy

Anthracyclines, widely used in paediatric cancer therapies for their high efficacy, are associated with dose-dependent cardiotoxicity. This contributes to an increasing burden of cancer therapy–related cardiovascular disease, particularly as 5-year survival rates for childhood leukaemia and long-term life expectancy continue to improve [61, 62]. The cardiotoxic effects of anthracyclines typically manifest as cardiomyopathy/left ventricular dysfunction [63]. Childhood cancer survivors face a 5–15-fold elevated incidence of congestive heart failure compared to the general population [64]. The American College of Sports Medicine and the American Cancer Society, supported by the ACC/AHA, advise that cancer survivors should “avoid inactivity” and perform 150 min of moderate- or 75 min of vigorous-intensity aerobic activity weekly, along with two strength training sessions, tailored to individual needs [65]. Although higher levels of physical exercise are linked to lower risk of cardiovascular disease and mortality, evidence on physical activity as a cardioprotective therapy is insufficient [66, 67]. This has led to the term “exercise cardio-oncology” to address this gap for potential cardioprotective effects of exercise in patients who have received anthracyclines [68]. Recent studies demonstrate that individually prescribed aerobic exercise is safe, positively influences left ventricular function, and enhances cardiopulmonary fitness and muscular strength and everyday physical activity in survivors under 18 years of age [69–72]. The AHA currently recommends a traditional cardiac rehabilitation approach for childhood cancer survivors delivered by an exercise professional with specific cancer and exercise knowledge [62].

## Acute myocarditis

Myocarditis is a leading cause of SCD in children and young athletes [73, 74] regardless of myocardial inflammation severity [76]. Animal models have shown that exertion during active disease may worsen myocardial injury, and relapses after early return to sport have been reported [76], underscoring the need for strict RTP protocols. Current recommendations are based on case series and low-quality cohort studies; however, consensus supports complete restriction from physical activity for at least 3–6 months, depending on disease severity, LV function, and extent of myocardial involvement. RTP requires normalised cardiac biomarkers, recovered LV function, and absence of complex arrhythmias on 24-h Holter and exercise testing [77–80]. In a paediatric myocarditis registry, the recommended time away from training ranged from 4 weeks to 1 year. Reassuringly, among those who returned to sport, recurrence was rare (3.4%) [76]. Persistent LGE on CMR may increase arrhythmic risk [81–85] though its significance in athletes remains unclear due to possible physiological remodelling [86, 87] The need for repeat CMR is debated: while not mandated by AHA/ACC guidelines [83] it is recommended by the ESC [13] to exclude active inflammation. Athletes with > 20% LGE or persistent dysfunction should abstain from sport [13] but RTP with residual scar may be considered case-by-case, if asymptomatic and arrhythmia-free [13, 81, 82]. Regular follow-up is advised, especially during the first 2 years post-diagnosis [86, 87]. See Table 2 for suggested workup, contraindications and follow-up.

## Aortopathies/heritable aortic diseases

Heritable thoracic aortic diseases (HTAD) in children are rare, with Marfan syndrome (MFS) and Loeys-Dietz syndrome (LDS) representing the most clinically relevant entities. Despite their potential for life-threatening complications, evidence regarding the role and safety of physical activity in children with HTAD remains limited [88]. The primary concerns associated with exercise in paediatric HTAD include the risk of aortic dissection (AoD) due to exercise-induced increases in blood pressure and aortic wall stress and the potential for accelerated progression of aortic dilation [88]. However, data supporting these risks in paediatric HTAD populations are scarce. The overall incidence of AoD in paediatric HTAD is generally low [89]. Reports of exercise-induced AoD in this population are limited to isolated case reports, predominantly involving adults. When extrapolating from large cohorts of paediatric athletes, the incidence of aortic dissection appears to be low, suggesting that the absolute risk in this age group may be minimal [90]. Similarly, data regarding exercise related progression of aortic dimensions in children with HTAD are virtually absent. Observational studies in young athletes have identified mild aortic dilation in a small subset of participants [90]. Although elite athletes have been shown to exhibit slightly larger aortic diameters, these differences were clinically negligible and were confounded by limitations in body surface area (BSA) matching and other methodological considerations [91]. However, the impact of exercise on aortic dimensions may differ in children with connective tissue disorders compared to healthy athletic populations. In a small cohort of young patients with HTAD (mean age 26 years), no association was found between aortic disease severity and either lifetime exercise volume or participation in competitive sports [92]. Interestingly, preclinical studies in murine models of MFS have demonstrated that moderate aerobic exercise may attenuate the rate of aortic growth and reduce aortic elastic fibre fragmentation [93]. In paediatric patients with MFS, data from lifestyle interventions—such as daily step-count programmes targeting 10,000 steps/day—have similarly suggested a potential benefit in reducing the rate of aortic dilation [94]. Children with HTAD often exhibit significantly reduced cardiorespiratory fitness, accompanied by marked chronotropic incompetence during exercise. This impairment in physical fitness has been associated with a diminished quality of life [95]. Moreover, higher levels of physical activity have been linked to improved quality of life in young patients with HTAD [92]. Emerging evidence indicates that tailored exercise interventions can improve physical fitness in both paediatric and adult HTAD populations and may even improve aortic dilation [94, 96]. Paediatric guidelines for physical activity in HTAD are generally less restrictive than adult recommendations (see Table 3). Across all age groups, competitive sports are generally discouraged—excluding skill-based or low intensity activities—with exceptions made through SDM ([23]. For children under 10 years of age, virtually no additional restrictions are advised. Above the age of 10, recommendations are based on the type of sport (e.g. avoidance of intense isometric sports or sports involving blunt trauma to the chest, e.g. rugby) and the severity of aortic involvement. Specifically, individuals with an aortic root z-score ≥ 3 or diameter ≥ 40 mm are limited to moderate-intensity activities [13, 97]. Despite these recommendations, significant variability exists in adherence at both the clinician and patient level [92]. Notably, up to 65% of young patients with HTAD report participating in competitive sports despite advised restrictions [92].

**Table 3:** Suggested recommendations to allow sports participation, contraindications, and follow-up in aortopathies (adapted from ESC guidelines on sports cardiology and exercise in patients with cardiovascular disease) ([13]

Condition	Assessment before sport	Sport contraindication	Follow-up
Marfan syndrome/Loeys-Dietz syndrome	Echocardiography CT/CMR	• Static sports • competitive sports in patients with severe aortic dilatation (> 45 mm) or *Z* score >/= 4	6–12 months depending on aortic dilation

*CT*, computed tomography;* MRI*, magnetic resonance imaging

## Competitive sports with an ICD

The ESC advises that ICDs should not be considered a substitute for sports restrictions in cases of severe disease and specifically recommends the avoidance of all collision sports [13]. The AHA/ACC suggests that competitive sports may be reasonable, provided there is rhythm stability and robust SDM regarding the individual’s underlying condition and the potential risks associated with device malfunction during contact sports [23]. Registry data from 129 young athletes (under 21 years) with ICDs reported that while shocks were common—27% of participants experienced at least one—only one significant arrhythmia occurred during competition [98]. More recent findings from a cohort of 125 athletes with ICDs and genetic heart conditions demonstrated that although breakthrough cardiac events (BCEs) were frequent (6.3 per 100 patient-years), there were no deaths or sports-related ICD malfunctions. Most BCEs were appropriate ICD shocks, half of which occurred outside of sporting activity [99]. Optimisation of ICD programming using a high-rate cutoff and long detection durations has been shown to reduce the incidence of shocks without compromising survival or syncope outcomes [100]. It would seem prudent to advise against sports at elevated risk of collision, e.g. contact sports and rock climbing, due to the risk of ICD damage [101].

## Shared decision-making (paediatric considerations)

**Fig. 1:**
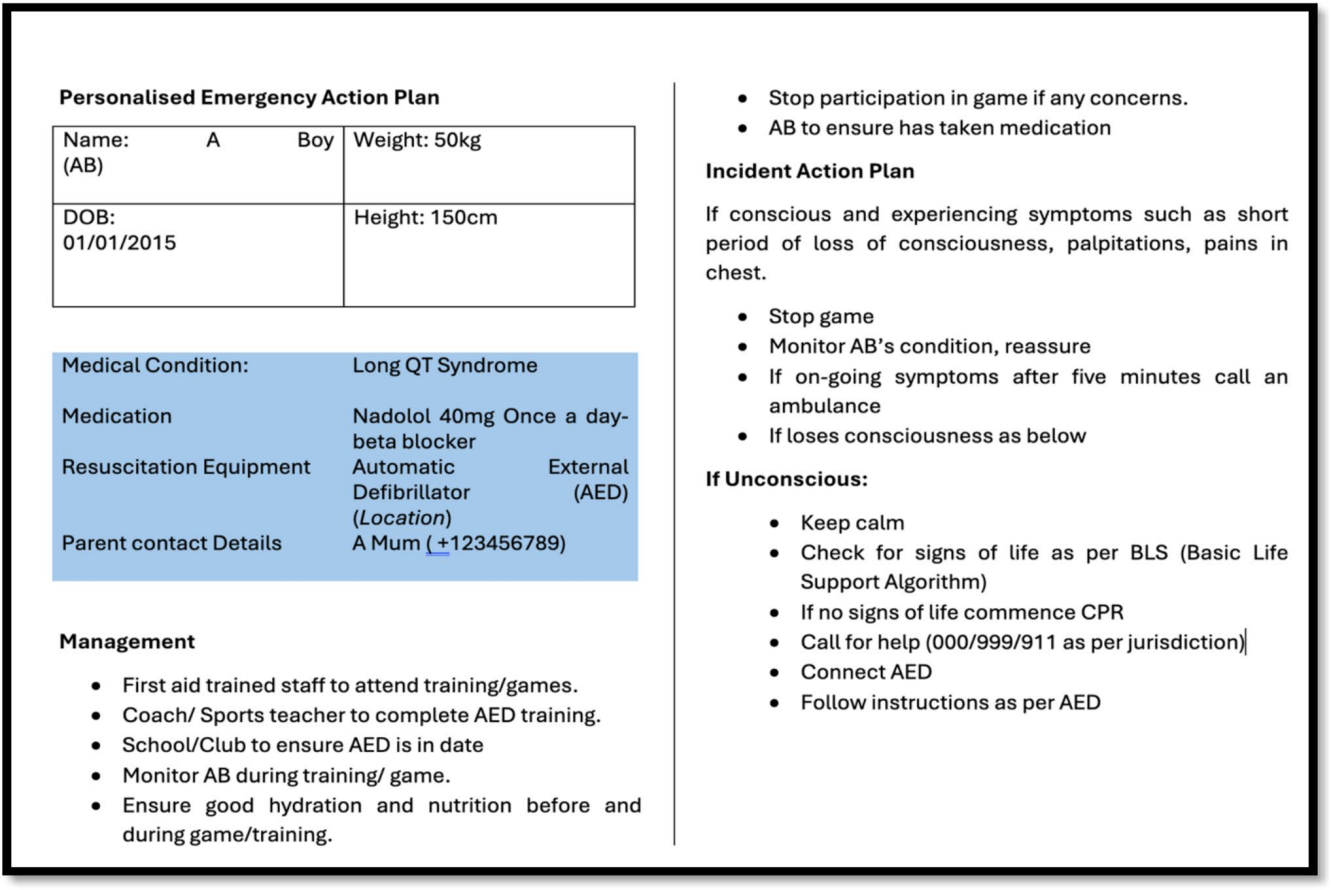
Example of a personalised emergency action plan for a hypothetical patient who is ten and wants to engage in sports at school. AED, automatic external defibrillator; CPR, cardiopulmonary resuscitation

Shared decision-making regarding the risks associated with sports participation in adults with inherited cardiac conditions is a complex process that requires expert guidance from specialists in the field. This approach has been successfully implemented in specialist centres [14, 15, 19, 102]. However, applying this model to children and their parents presents additional challenges. Factors such as Gillick competence, peer influence, parental aspirations, sporting ambitions, underlying anxieties and risk tolerance, and any perceived power imbalance between the clinician and the parents may all influence decision-making [14, 102]. In addition, families are often non-compliant with guidelines, whether purposefully or otherwise [25, 103]. Clinicians must approach these discussions with sensitivity, ensuring that communication is developmentally appropriate and tailored to the needs of both the child and their family. A careful balance must be struck between the (often small) risks—such as syncope, sudden cardiac death, disease progression, or the psychological burden of risk associated with physical activity—and the substantial benefits of regular exercise, including improved mental health, self-esteem, physical fitness, metabolic control, cardiovascular and pulmonary function, and overall well-being [104–106]. Legal considerations regarding liability in the event of an event are also understandable clinician concerns, mitigated by clear communication with accurate documentation such as an exercise plan and emergency management plan, including training using a defibrillator [107]. On-going surveillance is vital following a RTP decision [15]. Utilisation of the wider MDT is vital in the inherited cardiac clinic including genetic counsellors, psychologists, and the nursing team to help manage patients. A personalised action plan for school and/or the sports club is advisable for individuals who do continue to compete (see Fig. 1). Due to the risk of distress if a young person is disqualified from competitive sports by their newly diagnosed condition, psychological support if desired should be offered [108].

## Future directions to improve evidence-based recommendations

Due to the rarity of ICCs, improving the evidence base requires multi-centre collaboration [21, 39, 109]. While randomised controlled trials (RCTs) aimed at increasing physical activity are desirable—and several pilot studies in children have been successfully conducted—these trials provide limited information [110, 111]. They offer insights into physical benefits and risks during the study period but are constrained by short follow-up durations [110–112]. Long-term outcomes are perhaps best addressed through condition-specific registries [113–115] and extended follow-up of observational studies [21] comparing patients engaging in sports with those who do not over their lifetime, in an analogous manner to the Framingham Heart Study [116, 117]. The dawn of targeted therapy for inherited cardiac conditions will no doubt have positive implications for these patients and the sporting activities they can safely engage in [43, 118].

## Conclusion

Children affected by ICCs, their parents, and the doctors advising them continue to face complex decisions regarding sports participation. In the more commonly encountered conditions (LQTS, HCM), emerging data support more individualised, risk-based approaches, especially in centres with specific expertise. The evidence base is far less robust in the rarer, more malignant conditions (such as CPVT) prompting cautious interpretation of guidelines. On-going research and disease specific registries and interdisciplinary collaboration are essential to refine recommendations and empower families to make informed choices around sports participation.

## Supplementary Information

Below is the link to the electronic supplementary material.

ESM 1 (PNG 418 KB)

## Data Availability

No datasets were generated or analysed during the current study.

## Abbreviations

ACC: American College of Cardiology
AHA: American Heart Association
ARVC: Arrhythmogenic right ventricular cardiomyopathy
BrS: Brugada syndrome
CMR: Cardiac magnetic resonance imaging
CM: Cardiomyopathy
CPVT: Catecholaminergic polymorphic ventricular tachycardia
CT: Computed tomography
DCM: Dilated cardiomyopathy
DSP: Desmoplakin
EAPC: European Association of Preventive Cardiology
ECG: Electrocardiogram
ESC: European Society of Cardiology
FLNC: Filamin C
HCM: Hypertrophic cardiomyopathy
HRS: Heart Rhythm Society
ICD: Implantable cardioverter defibrillator
ICC: Inherited cardiac condition
LCSD: Left cardiac sympathetic denervation
LGE: Late gadolinium enhancement
LMNA: Lamin A/C
LQTS: Long QT syndrome
LV: Left ventricle
LVOT: Left ventricular outflow tract
NDLVC: Non-dilated left ventricular cardiomyopathy
nSVT: Non-sustained ventricular tachycardia
PLN: Phospholamban
RBM20: RNA-binding motif protein 20
RTP: Return to play
RV: Right ventricle
SCA: Sudden cardiac arrest
SCD: Sudden cardiac death
SDM: Shared decision-making
TMEM43: Transmembrane 43
VA: Ventricular arrhythmia
