# Supplementary figures and images for: Physical activity and competitive sport safety for children affected by inherited cardiac conditions and selected acquired cardiomyopathies: emerging evidence and areas for further inquiry
Source: Eur J Pediatr. 2026 Mar 4;185(3):160. doi: 10.1007/s00431-026-06768-y (PMC12957645; doi:10.1007/s00431-026-06768-y)

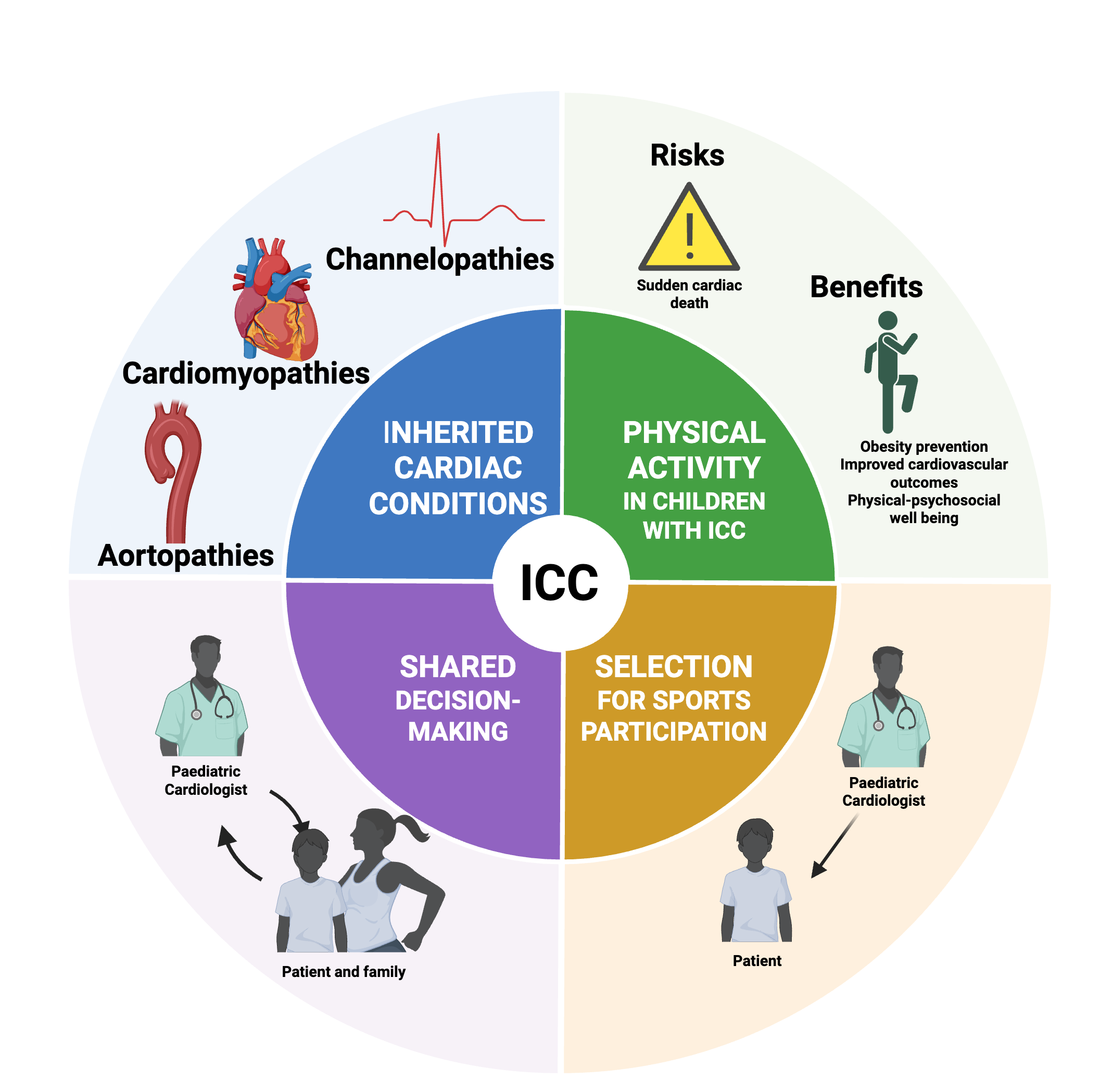

Supplement: Supplementary file 1 — (PNG 418 KB) [file 431_2026_6768_MOESM1_ESM.png]
